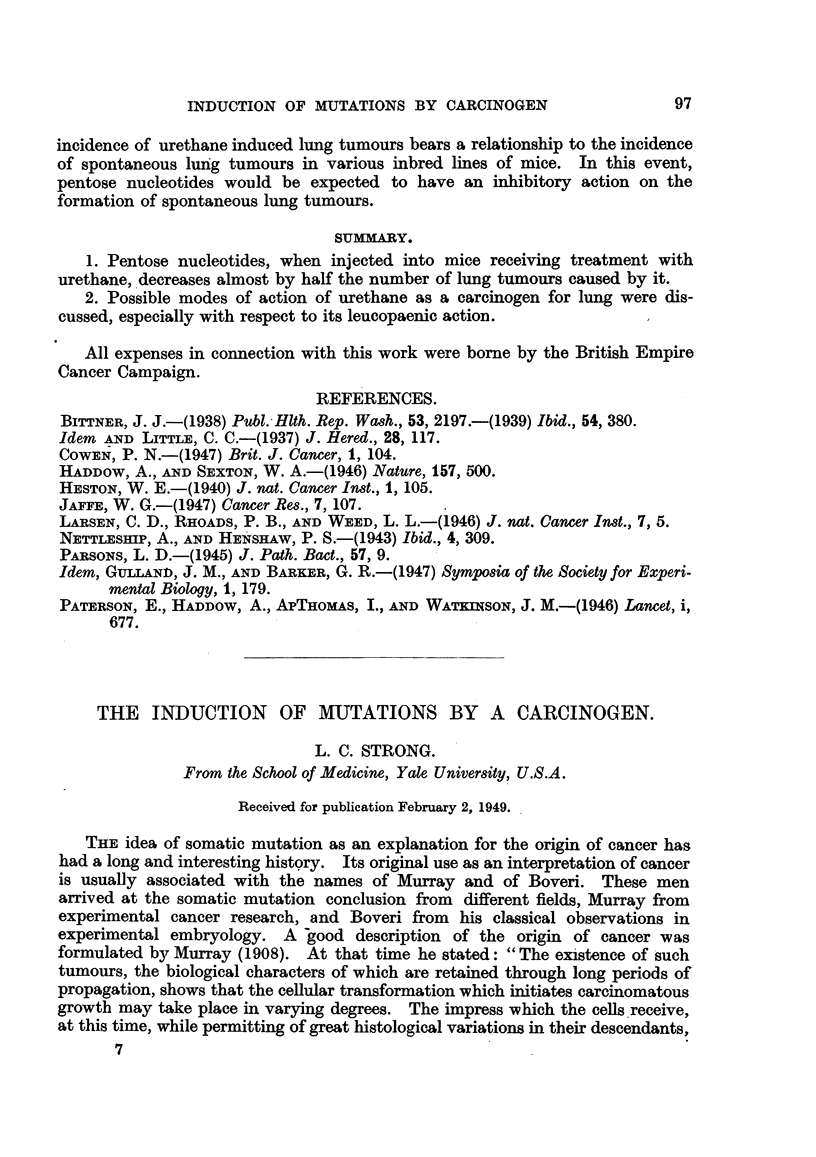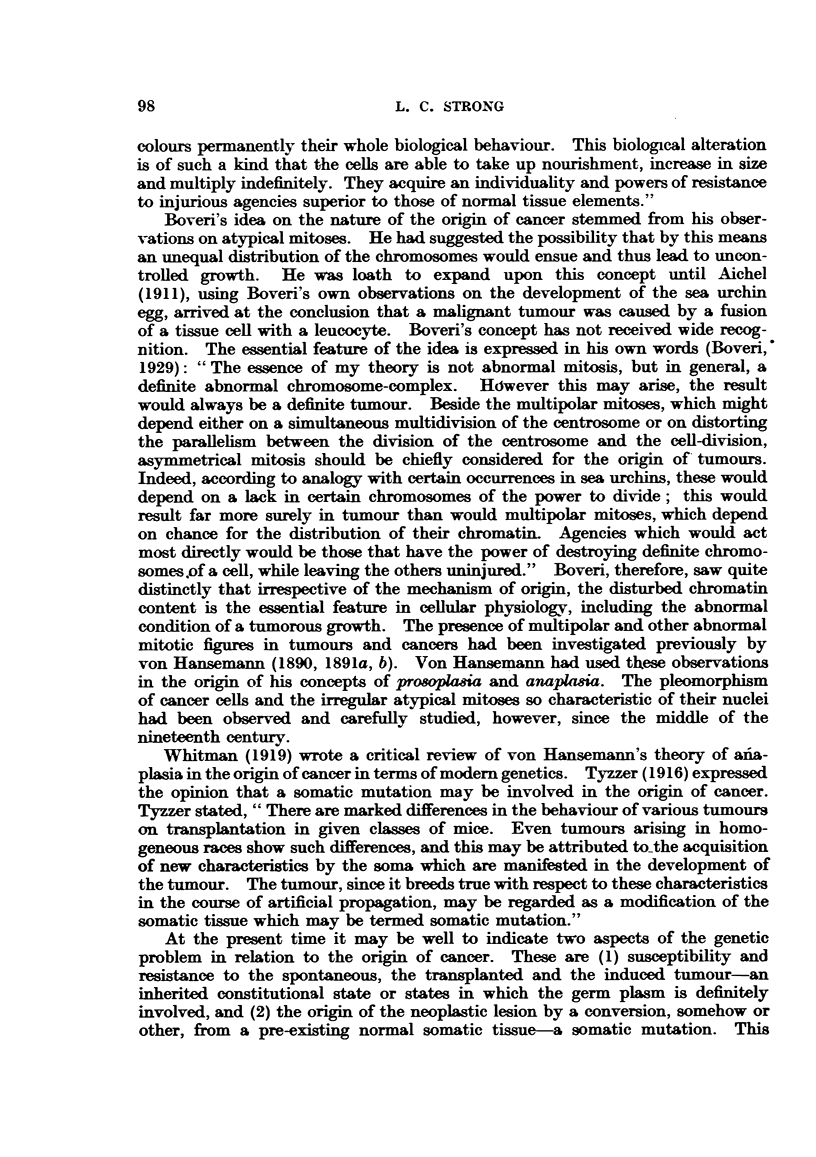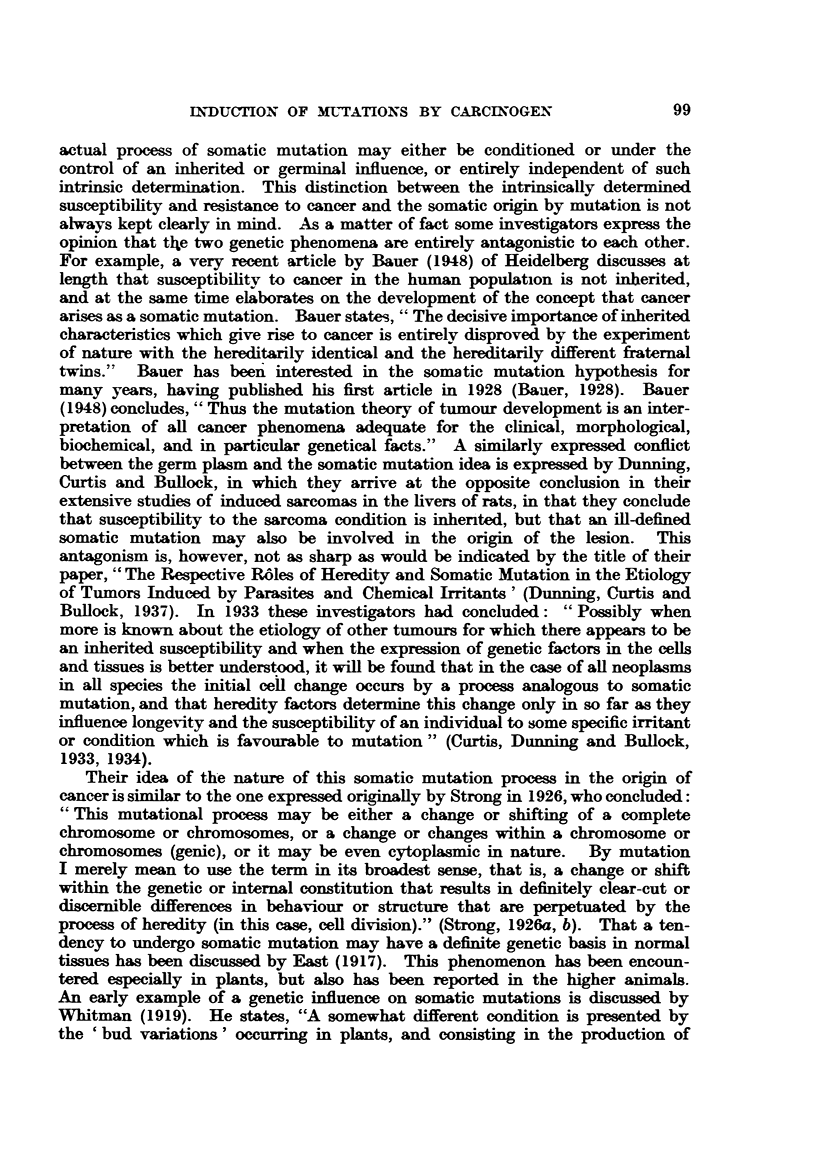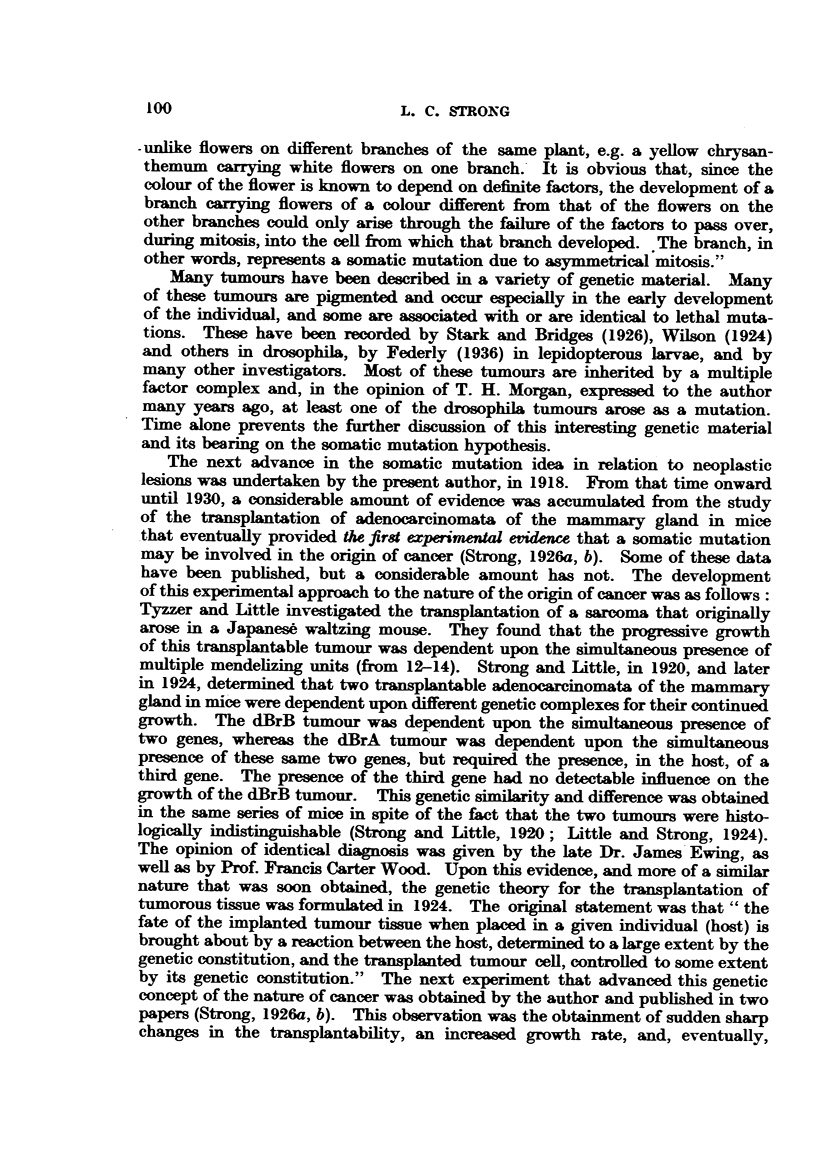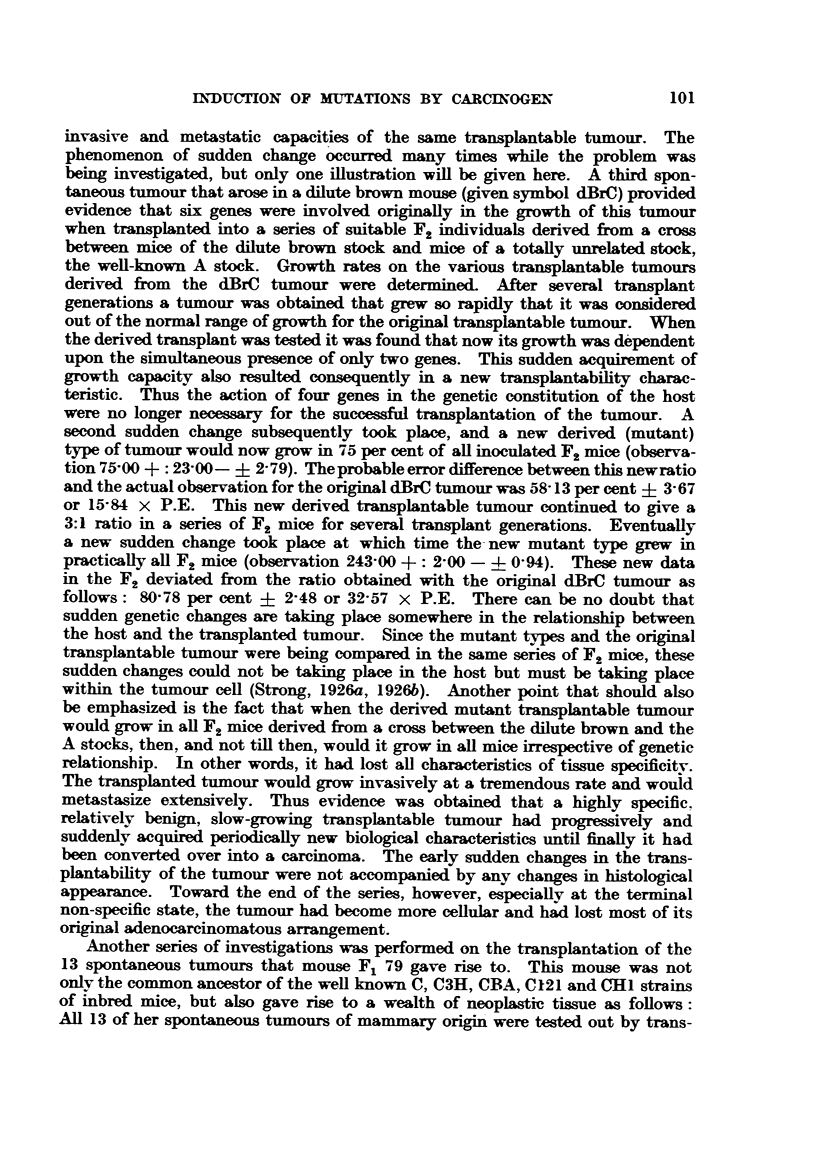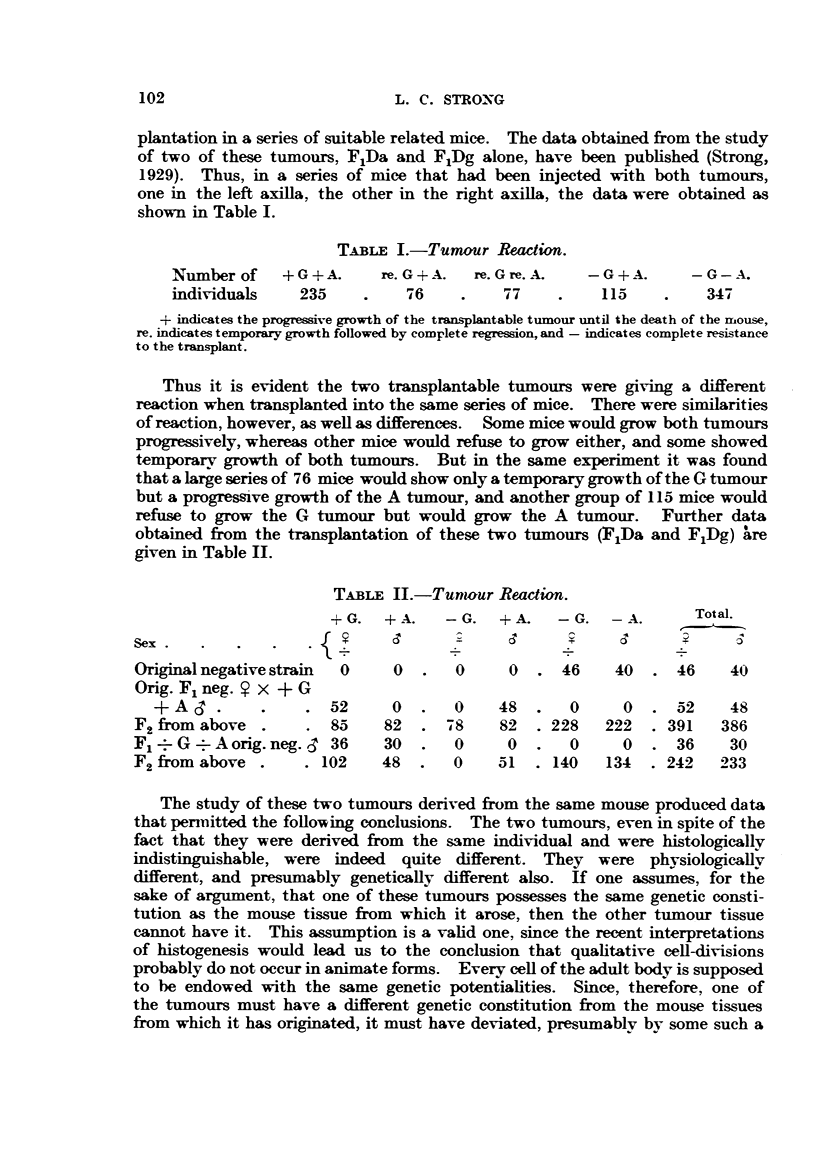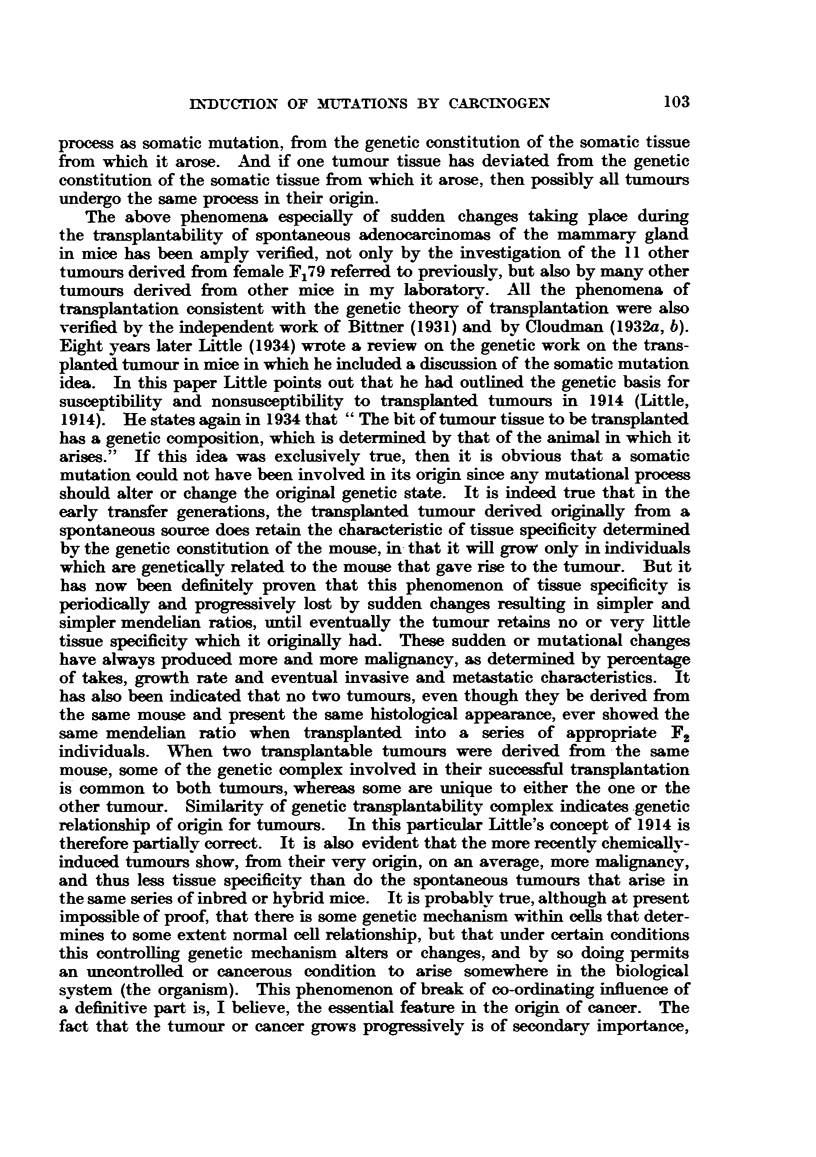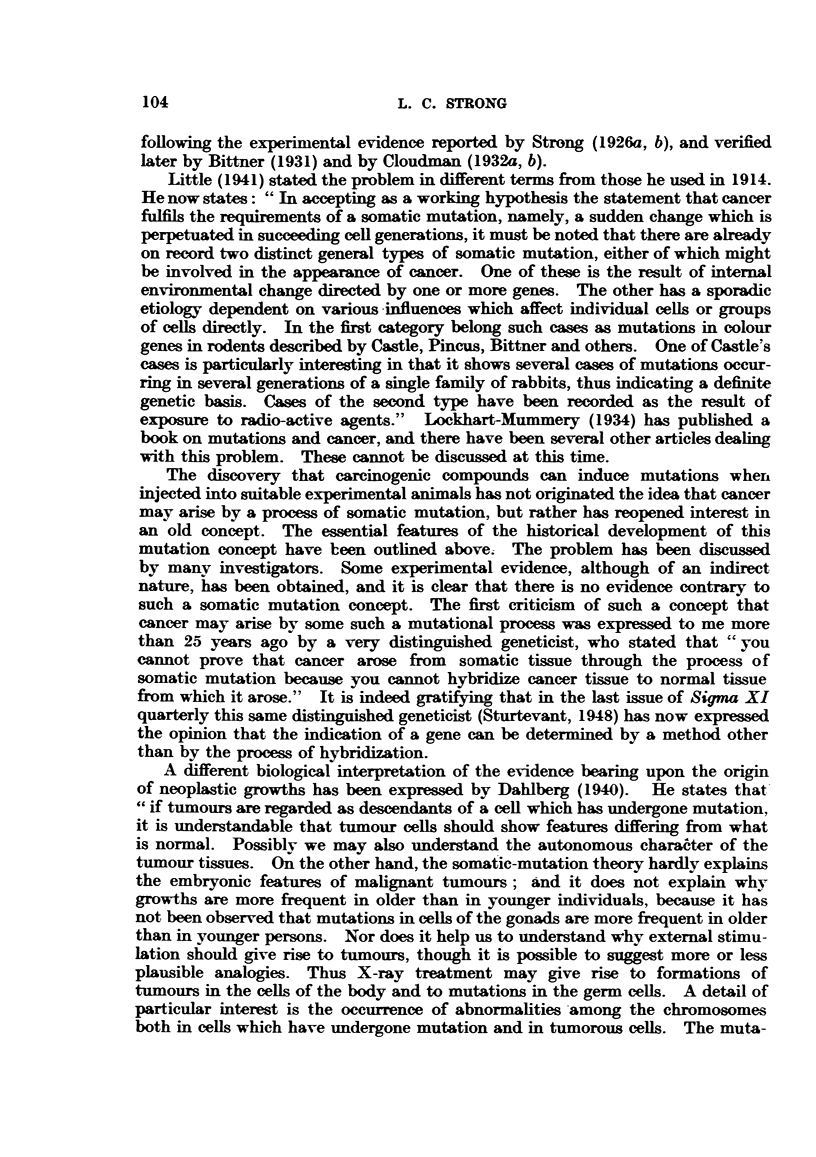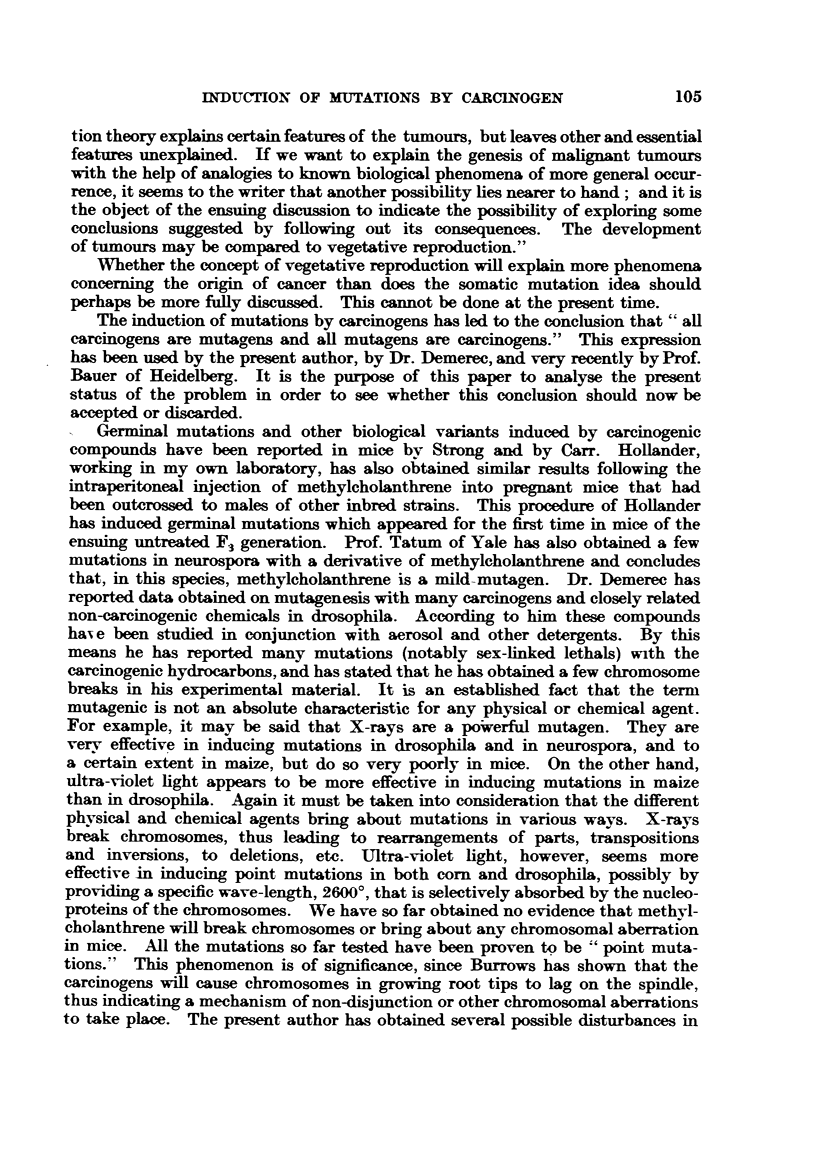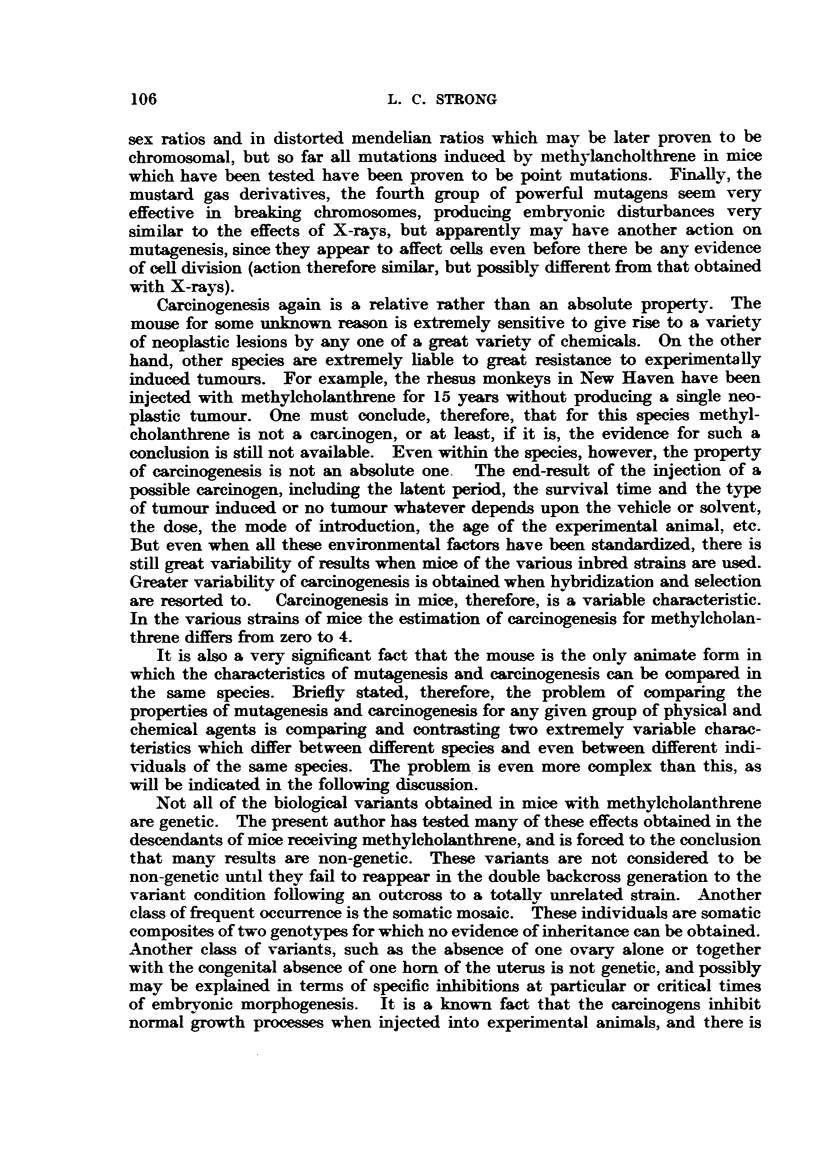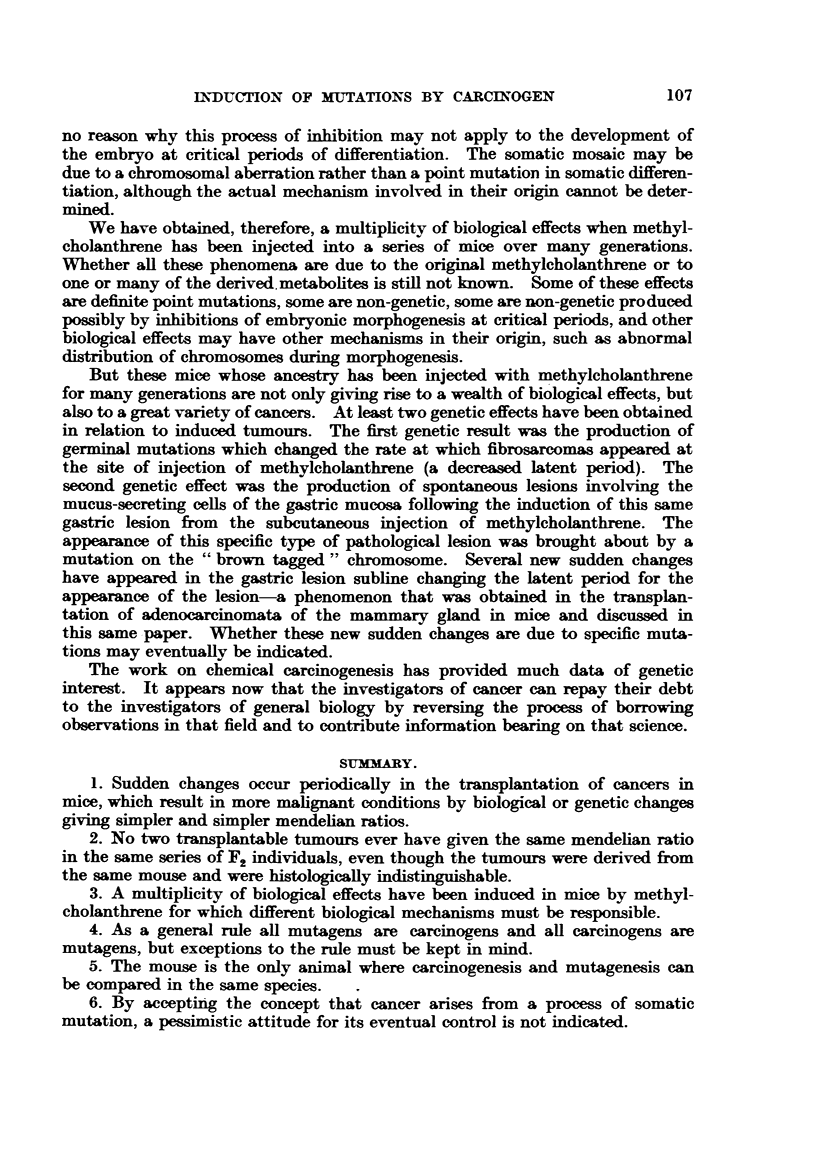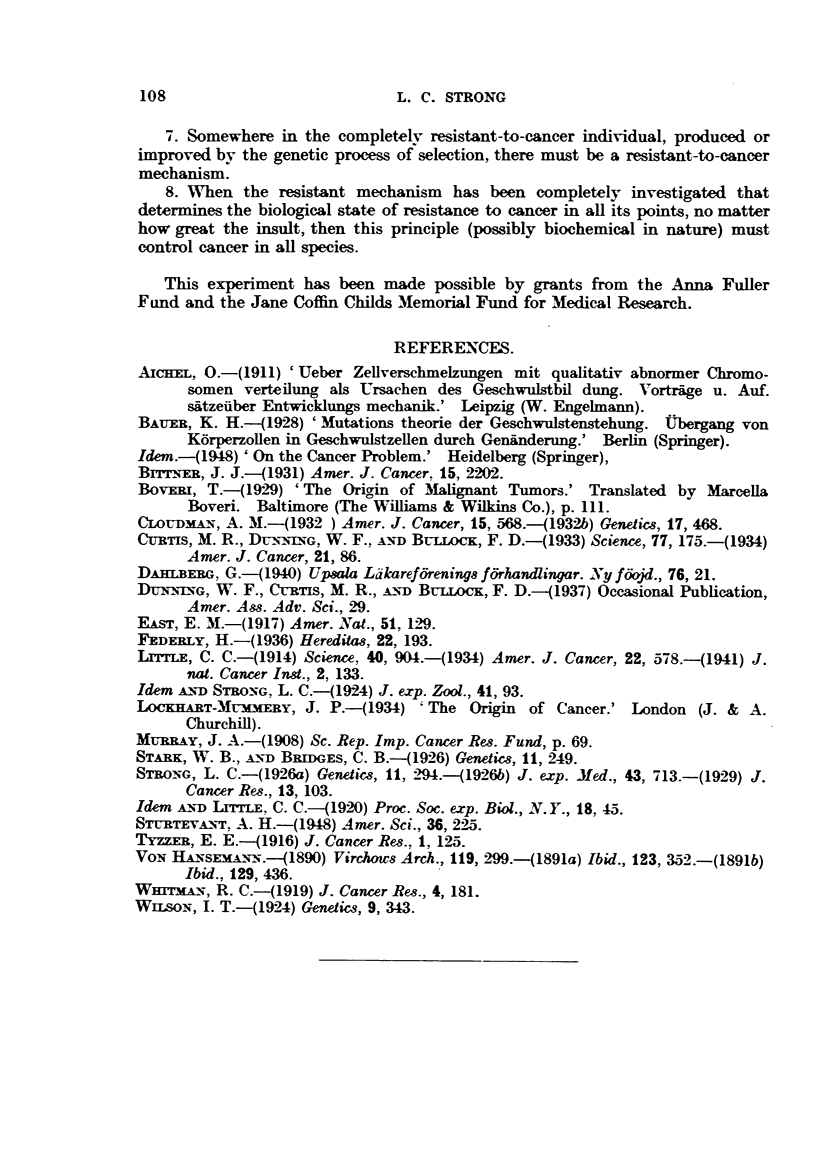# The Induction of Mutations by a Carcinogen

**DOI:** 10.1038/bjc.1949.12

**Published:** 1949-03

**Authors:** L. C. Strong


### THE INDUCTION OF MUTATIONS BY A CARCINOGEN.

L. C. STRONG.

*From the School of Medicine, Yale University, U.S.A.*

Received for publication February 2, 1949.

The idea of somatic mutation as an explanation for the origin of cancer has had a long and interesting history. Its original use as an interpretation of cancer is usually associated with the names of Murray and of Boveri. These men arrived at the somatic mutation conclusion from different fields, Murray from experimental cancer research, and Boveri from his classical observations in experimental embryology. A good description of the origin of cancer was formulated by Murray (1908). At that time he stated: "The existence of such tumours, the biological characters of which are retained through long periods of propagation, shows that the cellular transformation which initiates carcinomatous growth may take place in varying degrees. The impress which the cells receive, at this time, while permitting of great histological variations in their descendants, colours permanently their whole biological behaviour. This biological alteration is of such a kind that the cells are able to take up nourishment, increase in size and multiply indefinitely. They acquire an individuality and powers of resistance to injurious agencies superior to those of normal tissue elements."

Boveri's idea on the nature of the origin of cancer stemmed from his observations on atypical mitoses. He had suggested the possibility that by this means an unequal distribution of the chromosomes would ensue and thus lead to uncontrolled growth. He was loath to expand upon this concept until Aichel (1911), using Boveri's own observations on the development of the sea urchin egg, arrived at the conclusion that a malignant tumour was caused by a fusion of a tissue cell with a leucocyte. Boveri's concept has not received wide recognition. The essential feature of the idea is expressed in his own words (Boveri, 1929): "The essence of my theory is not abnormal mitosis, but in general, a definite abnormal chromosome-complex. However this may arise, the result would always be a definite tumour. Beside the multipolar mitoses, which might depend either on a simultaneous multidivision of the centrosome or on distorting the parallelism between the division of the centrosome and the cell-division, asymmetrical mitosis should be chiefly considered for the origin of tumours. Indeed, according to analogy with certain occurrences in sea urchins, these would depend on a lack in certain chromosomes of the power to divide; this would result far more surely in tumour than would multipolar mitoses, which depend on chance for the distribution of their chromatin. Agencies which would act most directly would be those that have the power of destroying definite chromosomes of a cell, while leaving the others uninjured." Boveri, therefore, saw quite distinctly that irrespective of the mechanism of origin, the disturbed chromatin content is the essential feature in cellular physiology, including the abnormal condition of a tumorous growth. The presence of multipolar and other abnormal mitotic figures in tumours and cancers had been investigated previously by von Hansemann (1890, 1891*a*, *b*). Von Hansemann had used these observations in the origin of his concepts of *prosoplasia* and *anaplasia*. The pleomorphism of cancer cells and the irregular atypical mitoses so characteristic of their nuclei had been observed and carefully studied, however, since the middle of the nineteenth century.

Whitman (1919) wrote a critical review of von Hansemann's theory of anaplasia in the origin of cancer in terms of modern genetics. Tyzzer (1916) expressed the opinion that a somatic mutation may be involved in the origin of cancer. Tyzzer stated, "There are marked differences in the behaviour of various tumours on transplantation in given classes of mice. Even tumours arising in homogeneous races show such differences, and this may be attributed to the acquisition of new characteristics by the soma which are manifested in the development of the tumour. The tumour, since it breeds true with respect to these characteristics in the course of artificial propagation, may be regarded as a modification of the somatic tissue which may be termed somatic mutation."

At the present time it may be well to indicate two aspects of the genetic problem in relation to the origin of cancer. These are (1) susceptibility and resistance to the spontaneous, the transplanted and the induced tumour—an inherited constitutional state or states in which the germ plasm is definitely involved, and (2) the origin of the neoplastic lesion by a conversion, somehow or other, from a pre-existing normal somatic tissue—a somatic mutation. This actual process of somatic mutation may either be conditioned or under the control of an inherited or germinal influence, or entirely independent of such intrinsic determination. This distinction between the intrinsically determined susceptibility and resistance to cancer and the somatic origin by mutation is not always kept clearly in mind. As a matter of fact some investigators express the opinion that the two genetic phenomena are entirely antagonistic to each other. For example, a very recent article by Bauer (1948) of Heidelberg discusses at length that susceptibility to cancer in the human population is not inherited, and at the same time elaborates on the development of the concept that cancer arises as a somatic mutation. Bauer states, " The decisive importance of inherited characteristics which give rise to cancer is entirely disproved by the experiment of nature with the hereditarily identical and the hereditarily different fraternal twins." Bauer has been interested in the somatic mutation hypothesis for many years, having published his first article in 1928 (Bauer, 1928). Bauer (1948) concludes, " Thus the mutation theory of tumour development is an interpretation of all cancer phenomena adequate for the clinical, morphological, biochemical, and in particular genetical facts." A similarly expressed conflict between the germ plasm and the somatic mutation idea is expressed by Dunning, Curtis and Bullock, in which they arrive at the opposite conclusion in their extensive studies of induced sarcomas in the livers of rats, in that they conclude that susceptibility to the sarcoma condition is inherited, but that an ill-defined somatic mutation may also be involved in the origin of the lesion. This antagonism is, however, not as sharp as would be indicated by the title of their paper, "The Respective Rôles of Heredity and Somatic Mutation in the Etiology of Tumors Induced by Parasites and Chemical Irritants ' (Dunning, Curtis and Bullock, 1937). In 1933 these investigators had concluded: " Possibly when more is known about the etiology of other tumours for which there appears to be an inherited susceptibility and when the expression of genetic factors in the cells and tissues is better understood, it will be found that in the case of all neoplasms in all species the initial cell change occurs by a process analogous to somatic mutation, and that heredity factors determine this change only in so far as they influence longevity and the susceptibility of an individual to some specific irritant or condition which is favourable to mutation " (Curtis, Dunning and Bullock, 1933, 1934).

Their idea of the nature of this somatic mutation process in the origin of cancer is similar to the one expressed originally by Strong in 1926, who concluded: " This mutational process may be either a change or shifting of a complete chromosome or chromosomes, or a change or changes within a chromosome or chromosomes (genic), or it may be even cytoplasmic in nature. By mutation I merely mean to use the term in its broadest sense, that is, a change or shift within the genetic or internal constitution that results in definitely clear-cut or discernible differences in behaviour or structure that are perpetuated by the process of heredity (in this case, cell division)." (Strong, 1926*a*, *b*). That a tendency to undergo somatic mutation may have a definite genetic basis in normal tissues has been discussed by East (1917). This phenomenon has been encountered especially in plants, but also has been reported in the higher animals. An early example of a genetic influence on somatic mutations is discussed by Whitman (1919). He states, "A somewhat different condition is presented by the ' bud variations ' occurring in plants, and consisting in the production of unlike flowers on different branches of the same plant, e.g. a yellow chrysanthemum carrying white flowers on one branch. It is obvious that, since the colour of the flower is known to depend on definite factors, the development of a branch carrying flowers of a colour different from that of the flowers on the other branches could only arise through the failure of the factors to pass over, during mitosis, into the cell from which that branch developed. The branch, in other words, represents a somatic mutation due to asymmetrical mitosis."

Many tumours have been described in a variety of genetic material. Many of these tumours are pigmented and occur especially in the early development of the individual, and some are associated with or are identical to lethal mutations. These have been recorded by Stark and Bridges (1926), Wilson (1924) and others in drosophila, by Federly (1936) in lepidopterous larvae, and by many other investigators. Most of these tumours are inherited by a multiple factor complex and, in the opinion of T. H. Morgan, expressed to the author many years ago, at least one of the drosophila tumours arose as a mutation. Time alone prevents the further discussion of this interesting genetic material and its bearing on the somatic mutation hypothesis.

The next advance in the somatic mutation idea in relation to neoplastic lesions was undertaken by the present author, in 1918. From that time onward until 1930, a considerable amount of evidence was accumulated from the study of the transplantation of adenocarcinomata of the mammary gland in mice that eventually provided *the first experimental evidence* that a somatic mutation may be involved in the origin of cancer (Strong, 1926*a*, *b*). Some of these data have been published, but a considerable amount has not. The development of this experimental approach to the nature of the origin of cancer was as follows: Tyzzer and Little investigated the transplantation of a sarcoma that originally arose in a Japanese waltzing mouse. They found that the progressive growth of this transplantable tumour was dependent upon the simultaneous presence of multiple mendelizing units (from 12–14). Strong and Little, in 1920, and later in 1924, determined that two transplantable adenocarcinomata of the mammary gland in mice were dependent upon different genetic complexes for their continued growth. The dBrB tumour was dependent upon the simultaneous presence of two genes, whereas the dBrA tumour was dependent upon the simultaneous presence of these same two genes, but required the presence, in the host, of a third gene. The presence of the third gene had no detectable influence on the growth of the dBrB tumour. This genetic similarity and difference was obtained in the same series of mice in spite of the fact that the two tumours were histologically indistinguishable (Strong and Little, 1920; Little and Strong, 1924). The opinion of identical diagnosis was given by the late Dr. James Ewing, as well as by Prof. Francis Carter Wood. Upon this evidence, and more of a similar nature that was soon obtained, the genetic theory for the transplantation of tumorous tissue was formulated in 1924. The original statement was that "the fate of the implanted tumour tissue when placed in a given individual (host) is brought about by a reaction between the host, determined to a large extent by the genetic constitution, and the transplanted tumour cell, controlled to some extent by its genetic constitution." The next experiment that advanced this genetic concept of the nature of cancer was obtained by the author and published in two papers (Strong, 1926*a*, *b*). This observation was the obtainment of sudden sharp changes in the transplantability, an increased growth rate, and, eventually, invasive and metastatic capacities of the same transplantable tumour. The phenomenon of sudden change occurred many times while the problem was being investigated, but only one illustration will be given here. A third spontaneous tumour that arose in a dilute brown mouse (given symbol dBrC) provided evidence that six genes were involved originally in the growth of this tumour when transplanted into a series of suitable $F_2$ individuals derived from a cross between mice of the dilute brown stock and mice of a totally unrelated stock, the well-known A stock. Growth rates on the various transplantable tumours derived from the dBrC tumour were determined. After several transplant generations a tumour was obtained that grew so rapidly that it was considered out of the normal range of growth for the original transplantable tumour. When the derived transplant was tested it was found that now its growth was dependent upon the simultaneous presence of only two genes. This sudden acquirement of growth capacity also resulted consequently in a new transplantability characteristic. Thus the action of four genes in the genetic constitution of the host were no longer necessary for the successful transplantation of the tumour. A second sudden change subsequently took place, and a new derived (mutant) type of tumour would now grow in 75 per cent of all inoculated $F_2$ mice (observation 75·00 + : 23·00 — $\pm$ 2·79). The probable error difference between this new ratio and the actual observation for the original dBrC tumour was 58·13 per cent $\pm$ 3·67 or 15·84 $\times$ P.E. This new derived transplantable tumour continued to give a 3:1 ratio in a series of $F_2$ mice for several transplant generations. Eventually a new sudden change took place at which time the new mutant type grew in practically all $F_2$ mice (observation 243·00 + : 2·00 — $\pm$ 0·94). These new data in the $F_2$ deviated from the ratio obtained with the original dBrC tumour as follows: 80·78 per cent $\pm$ 2·48 or 32·57 $\times$ P.E. There can be no doubt that sudden genetic changes are taking place somewhere in the relationship between the host and the transplanted tumour. Since the mutant types and the original transplantable tumour were being compared in the same series of $F_2$ mice, these sudden changes could not be taking place in the host but must be taking place within the tumour cell (Strong, 1926*a*, 1926*b*). Another point that should also be emphasized is the fact that when the derived mutant transplantable tumour would grow in all $F_2$ mice derived from a cross between the dilute brown and the A stocks, then, and not till then, would it grow in all mice irrespective of genetic relationship. In other words, it had lost all characteristics of tissue specificity. The transplanted tumour would grow invasively at a tremendous rate and would metastasize extensively. Thus evidence was obtained that a highly specific, relatively benign, slow-growing transplantable tumour had progressively and suddenly acquired periodically new biological characteristics until finally it had been converted over into a carcinoma. The early sudden changes in the transplantability of the tumour were not accompanied by any changes in histological appearance. Toward the end of the series, however, especially at the terminal non-specific state, the tumour had become more cellular and had lost most of its original adenocarcinomatous arrangement.

Another series of investigations was performed on the transplantation of the 13 spontaneous tumours that mouse $F_1$ 79 gave rise to. This mouse was not only the common ancestor of the well known C, C3H, CBA, C121 and CH1 strains of inbred mice, but also gave rise to a wealth of neoplastic tissue as follows: All 13 of her spontaneous tumours of mammary origin were tested out by transplantation in a series of suitable related mice. The data obtained from the study of two of these tumours, $F_1Da$ and $F_1Dg$ alone, have been published (Strong, 1929). Thus, in a series of mice that had been injected with both tumours, one in the left axilla, the other in the right axilla, the data were obtained as shown in Table I.

TABLE I.—*Tumour Reaction.*

| Number of individuals | + G + A. | re. G + A. | re. G re. A. | − G + A. | − G − A. |
|---|---|---|---|---|---|
| | 235 | 76 | 77 | 115 | 347 |

\+ indicates the progressive growth of the transplantable tumour until the death of the mouse, re. indicates temporary growth followed by complete regression, and — indicates complete resistance to the transplant.

Thus it is evident the two transplantable tumours were giving a different reaction when transplanted into the same series of mice. There were similarities of reaction, however, as well as differences. Some mice would grow both tumours progressively, whereas other mice would refuse to grow either, and some showed temporary growth of both tumours. But in the same experiment it was found that a large series of 76 mice would show only a temporary growth of the G tumour but a progressive growth of the A tumour, and another group of 115 mice would refuse to grow the G tumour but would grow the A tumour. Further data obtained from the transplantation of these two tumours ($F_1Da$ and $F_1Dg$) are given in Table II.

TABLE II.—*Tumour Reaction.*

| | + G. | + A. | − G. | + A. | − G. | − A. | Total. | |
|---|---|---|---|---|---|---|---|---|
| Sex | ♀ ÷ | ♂ | ♀ ÷ | ♂ | ♀ ÷ | ♂ | ♀ ÷ | ♂ |
| Original negative strain | 0 | 0 | 0 | 0 | 46 | 40 | 46 | 40 |
| Orig. $F_1$ neg. ♀ × + G + A ♂ | 52 | 0 | 0 | 48 | 0 | 0 | 52 | 48 |
| $F_2$ from above | 85 | 82 | 78 | 82 | 228 | 222 | 391 | 386 |
| $F_1$ ÷ G ÷ A orig. neg. ♂ | 36 | 30 | 0 | 0 | 0 | 0 | 36 | 30 |
| $F_2$ from above | 102 | 48 | 0 | 51 | 140 | 134 | 242 | 233 |

The study of these two tumours derived from the same mouse produced data that permitted the following conclusions. The two tumours, even in spite of the fact that they were derived from the same individual and were histologically indistinguishable, were indeed quite different. They were physiologically different, and presumably genetically different also. If one assumes, for the sake of argument, that one of these tumours possesses the same genetic constitution as the mouse tissue from which it arose, then the other tumour tissue cannot have it. This assumption is a valid one, since the recent interpretations of histogenesis would lead us to the conclusion that qualitative cell-divisions probably do not occur in animate forms. Every cell of the adult body is supposed to be endowed with the same genetic potentialities. Since, therefore, one of the tumours must have a different genetic constitution from the mouse tissues from which it has originated, it must have deviated, presumably by some such a process as somatic mutation, from the genetic constitution of the somatic tissue from which it arose. And if one tumour tissue has deviated from the genetic constitution of the somatic tissue from which it arose, then possibly all tumours undergo the same process in their origin.

The above phenomena especially of sudden changes taking place during the transplantability of spontaneous adenocarcinomas of the mammary gland in mice has been amply verified, not only by the investigation of the 11 other tumours derived from female $F_1$79 referred to previously, but also by many other tumours derived from other mice in my laboratory. All the phenomena of transplantation consistent with the genetic theory of transplantation were also verified by the independent work of Bittner (1931) and by Cloudman (1932*a*, *b*). Eight years later Little (1934) wrote a review on the genetic work on the transplanted tumour in mice in which he included a discussion of the somatic mutation idea. In this paper Little points out that he had outlined the genetic basis for susceptibility and nonsusceptibility to transplanted tumours in 1914 (Little, 1914). He states again in 1934 that " The bit of tumour tissue to be transplanted has a genetic composition, which is determined by that of the animal in which it arises." If this idea was exclusively true, then it is obvious that a somatic mutation could not have been involved in its origin since any mutational process should alter or change the original genetic state. It is indeed true that in the early transfer generations, the transplanted tumour derived originally from a spontaneous source does retain the characteristic of tissue specificity determined by the genetic constitution of the mouse, in that it will grow only in individuals which are genetically related to the mouse that gave rise to the tumour. But it has now been definitely proven that this phenomenon of tissue specificity is periodically and progressively lost by sudden changes resulting in simpler and simpler mendelian ratios, until eventually the tumour retains no or very little tissue specificity which it originally had. These sudden or mutational changes have always produced more and more malignancy, as determined by percentage of takes, growth rate and eventual invasive and metastatic characteristics. It has also been indicated that no two tumours, even though they be derived from the same mouse and present the same histological appearance, ever showed the same mendelian ratio when transplanted into a series of appropriate $F_2$ individuals. When two transplantable tumours were derived from the same mouse, some of the genetic complex involved in their successful transplantation is common to both tumours, whereas some are unique to either the one or the other tumour. Similarity of genetic transplantability complex indicates genetic relationship of origin for tumours. In this particular Little's concept of 1914 is therefore partially correct. It is also evident that the more recently chemically-induced tumours show, from their very origin, on an average, more malignancy, and thus less tissue specificity than do the spontaneous tumours that arise in the same series of inbred or hybrid mice. It is probably true, although at present impossible of proof, that there is some genetic mechanism within cells that determines to some extent normal cell relationship, but that under certain conditions this controlling genetic mechanism alters or changes, and by so doing permits an uncontrolled or cancerous condition to arise somewhere in the biological system (the organism). This phenomenon of break of co-ordinating influence of a definitive part is, I believe, the essential feature in the origin of cancer. The fact that the tumour or cancer grows progressively is of secondary importance, following the experimental evidence reported by Strong (1926*a*, *b*), and verified later by Bittner (1931) and by Cloudman (1932*a*, *b*).

Little (1941) stated the problem in different terms from those he used in 1914. He now states: "In accepting as a working hypothesis the statement that cancer fulfils the requirements of a somatic mutation, namely, a sudden change which is perpetuated in succeeding cell generations, it must be noted that there are already on record two distinct general types of somatic mutation, either of which might be involved in the appearance of cancer. One of these is the result of internal environmental change directed by one or more genes. The other has a sporadic etiology dependent on various influences which affect individual cells or groups of cells directly. In the first category belong such cases as mutations in colour genes in rodents described by Castle, Pincus, Bittner and others. One of Castle's cases is particularly interesting in that it shows several cases of mutations occurring in several generations of a single family of rabbits, thus indicating a definite genetic basis. Cases of the second type have been recorded as the result of exposure to radio-active agents." Lockhart-Mummery (1934) has published a book on mutations and cancer, and there have been several other articles dealing with this problem. These cannot be discussed at this time.

The discovery that carcinogenic compounds can induce mutations when injected into suitable experimental animals has not originated the idea that cancer may arise by a process of somatic mutation, but rather has reopened interest in an old concept. The essential features of the historical development of this mutation concept have been outlined above. The problem has been discussed by many investigators. Some experimental evidence, although of an indirect nature, has been obtained, and it is clear that there is no evidence contrary to such a somatic mutation concept. The first criticism of such a concept that cancer may arise by some such a mutational process was expressed to me more than 25 years ago by a very distinguished geneticist, who stated that "you cannot prove that cancer arose from somatic tissue through the process of somatic mutation because you cannot hybridize cancer tissue to normal tissue from which it arose." It is indeed gratifying that in the last issue of *Sigma XI* quarterly this same distinguished geneticist (Sturtevant, 1948) has now expressed the opinion that the indication of a gene can be determined by a method other than by the process of hybridization.

A different biological interpretation of the evidence bearing upon the origin of neoplastic growths has been expressed by Dahlberg (1940). He states that "if tumours are regarded as descendants of a cell which has undergone mutation, it is understandable that tumour cells should show features differing from what is normal. Possibly we may also understand the autonomous character of the tumour tissues. On the other hand, the somatic-mutation theory hardly explains the embryonic features of malignant tumours; and it does not explain why growths are more frequent in older than in younger individuals, because it has not been observed that mutations in cells of the gonads are more frequent in older than in younger persons. Nor does it help us to understand why external stimulation should give rise to tumours, though it is possible to suggest more or less plausible analogies. Thus X-ray treatment may give rise to formations of tumours in the cells of the body and to mutations in the germ cells. A detail of particular interest is the occurrence of abnormalities among the chromosomes both in cells which have undergone mutation and in tumorous cells. The mutation theory explains certain features of the tumours, but leaves other and essential features unexplained. If we want to explain the genesis of malignant tumours with the help of analogies to known biological phenomena of more general occurrence, it seems to the writer that another possibility lies nearer to hand ; and it is the object of the ensuing discussion to indicate the possibility of exploring some conclusions suggested by following out its consequences. The development of tumours may be compared to vegetative reproduction."

Whether the concept of vegetative reproduction will explain more phenomena concerning the origin of cancer than does the somatic mutation idea should perhaps be more fully discussed. This cannot be done at the present time.

The induction of mutations by carcinogens has led to the conclusion that "all carcinogens are mutagens and all mutagens are carcinogens." This expression has been used by the present author, by Dr. Demerec, and very recently by Prof. Bauer of Heidelberg. It is the purpose of this paper to analyse the present status of the problem in order to see whether this conclusion should now be accepted or discarded.

Germinal mutations and other biological variants induced by carcinogenic compounds have been reported in mice by Strong and by Carr. Hollander, working in my own laboratory, has also obtained similar results following the intraperitoneal injection of methylcholanthrene into pregnant mice that had been outcrossed to males of other inbred strains. This procedure of Hollander has induced germinal mutations which appeared for the first time in mice of the ensuing untreated $F_3$ generation. Prof. Tatum of Yale has also obtained a few mutations in neurospora with a derivative of methylcholanthrene and concludes that, in this species, methylcholanthrene is a mild-mutagen. Dr. Demerec has reported data obtained on mutagenesis with many carcinogens and closely related non-carcinogenic chemicals in drosophila. According to him these compounds have been studied in conjunction with aerosol and other detergents. By this means he has reported many mutations (notably sex-linked lethals) with the carcinogenic hydrocarbons, and has stated that he has obtained a few chromosome breaks in his experimental material. It is an established fact that the term mutagenic is not an absolute characteristic for any physical or chemical agent. For example, it may be said that X-rays are a powerful mutagen. They are very effective in inducing mutations in drosophila and in neurospora, and to a certain extent in maize, but do so very poorly in mice. On the other hand, ultra-violet light appears to be more effective in inducing mutations in maize than in drosophila. Again it must be taken into consideration that the different physical and chemical agents bring about mutations in various ways. X-rays break chromosomes, thus leading to rearrangements of parts, transpositions and inversions, to deletions, etc. Ultra-violet light, however, seems more effective in inducing point mutations in both corn and drosophila, possibly by providing a specific wave-length, 2600°, that is selectively absorbed by the nucleoproteins of the chromosomes. We have so far obtained no evidence that methylcholanthrene will break chromosomes or bring about any chromosomal aberration in mice. All the mutations so far tested have been proven to be "point mutations." This phenomenon is of significance, since Burrows has shown that the carcinogens will cause chromosomes in growing root tips to lag on the spindle, thus indicating a mechanism of non-disjunction or other chromosomal aberrations to take place. The present author has obtained several possible disturbances in sex ratios and in distorted mendelian ratios which may be later proven to be chromosomal, but so far all mutations induced by methylancholthrene in mice which have been tested have been proven to be point mutations. Finally, the mustard gas derivatives, the fourth group of powerful mutagens seem very effective in breaking chromosomes, producing embryonic disturbances very similar to the effects of X-rays, but apparently may have another action on mutagenesis, since they appear to affect cells even before there be any evidence of cell division (action therefore similar, but possibly different from that obtained with X-rays).

Carcinogenesis again is a relative rather than an absolute property. The mouse for some unknown reason is extremely sensitive to give rise to a variety of neoplastic lesions by any one of a great variety of chemicals. On the other hand, other species are extremely liable to great resistance to experimentally induced tumours. For example, the rhesus monkeys in New Haven have been injected with methylcholanthrene for 15 years without producing a single neoplastic tumour. One must conclude, therefore, that for this species methylcholanthrene is not a carcinogen, or at least, if it is, the evidence for such a conclusion is still not available. Even within the species, however, the property of carcinogenesis is not an absolute one. The end-result of the injection of a possible carcinogen, including the latent period, the survival time and the type of tumour induced or no tumour whatever depends upon the vehicle or solvent, the dose, the mode of introduction, the age of the experimental animal, etc. But even when all these environmental factors have been standardized, there is still great variability of results when mice of the various inbred strains are used. Greater variability of carcinogenesis is obtained when hybridization and selection are resorted to. Carcinogenesis in mice, therefore, is a variable characteristic. In the various strains of mice the estimation of carcinogenesis for methylcholanthrene differs from zero to 4.

It is also a very significant fact that the mouse is the only animate form in which the characteristics of mutagenesis and carcinogenesis can be compared in the same species. Briefly stated, therefore, the problem of comparing the properties of mutagenesis and carcinogenesis for any given group of physical and chemical agents is comparing and contrasting two extremely variable characteristics which differ between different species and even between different individuals of the same species. The problem is even more complex than this, as will be indicated in the following discussion.

Not all of the biological variants obtained in mice with methylcholanthrene are genetic. The present author has tested many of these effects obtained in the descendants of mice receiving methylcholanthrene, and is forced to the conclusion that many results are non-genetic. These variants are not considered to be non-genetic until they fail to reappear in the double backcross generation to the variant condition following an outcross to a totally unrelated strain. Another class of frequent occurrence is the somatic mosaic. These individuals are somatic composites of two genotypes for which no evidence of inheritance can be obtained. Another class of variants, such as the absence of one ovary alone or together with the congenital absence of one horn of the uterus is not genetic, and possibly may be explained in terms of specific inhibitions at particular or critical times of embryonic morphogenesis. It is a known fact that the carcinogens inhibit normal growth processes when injected into experimental animals, and there is no reason why this process of inhibition may not apply to the development of the embryo at critical periods of differentiation. The somatic mosaic may be due to a chromosomal aberration rather than a point mutation in somatic differentiation, although the actual mechanism involved in their origin cannot be determined.

We have obtained, therefore, a multiplicity of biological effects when methylcholanthrene has been injected into a series of mice over many generations. Whether all these phenomena are due to the original methylcholanthrene or to one or many of the derived metabolites is still not known. Some of these effects are definite point mutations, some are non-genetic, some are non-genetic produced possibly by inhibitions of embryonic morphogenesis at critical periods, and other biological effects may have other mechanisms in their origin, such as abnormal distribution of chromosomes during morphogenesis.

But these mice whose ancestry has been injected with methylcholanthrene for many generations are not only giving rise to a wealth of biological effects, but also to a great variety of cancers. At least two genetic effects have been obtained in relation to induced tumours. The first genetic result was the production of germinal mutations which changed the rate at which fibrosarcomas appeared at the site of injection of methylcholanthrene (a decreased latent period). The second genetic effect was the production of spontaneous lesions involving the mucus-secreting cells of the gastric mucosa following the induction of this same gastric lesion from the subcutaneous injection of methylcholanthrene. The appearance of this specific type of pathological lesion was brought about by a mutation on the " brown tagged " chromosome. Several new sudden changes have appeared in the gastric lesion subline changing the latent period for the appearance of the lesion—a phenomenon that was obtained in the transplantation of adenocarcinomata of the mammary gland in mice and discussed in this same paper. Whether these new sudden changes are due to specific mutations may eventually be indicated.

The work on chemical carcinogenesis has provided much data of genetic interest. It appears now that the investigators of cancer can repay their debt to the investigators of general biology by reversing the process of borrowing observations in that field and to contribute information bearing on that science.

### SUMMARY.

1. Sudden changes occur periodically in the transplantation of cancers in mice, which result in more malignant conditions by biological or genetic changes giving simpler and simpler mendelian ratios.

2. No two transplantable tumours ever have given the same mendelian ratio in the same series of $F_2$ individuals, even though the tumours were derived from the same mouse and were histologically indistinguishable.

3. A multiplicity of biological effects have been induced in mice by methylcholanthrene for which different biological mechanisms must be responsible.

4. As a general rule all mutagens are carcinogens and all carcinogens are mutagens, but exceptions to the rule must be kept in mind.

5. The mouse is the only animal where carcinogenesis and mutagenesis can be compared in the same species.

6. By accepting the concept that cancer arises from a process of somatic mutation, a pessimistic attitude for its eventual control is not indicated.

7. Somewhere in the completely resistant-to-cancer individual, produced or improved by the genetic process of selection, there must be a resistant-to-cancer mechanism.

8. When the resistant mechanism has been completely investigated that determines the biological state of resistance to cancer in all its points, no matter how great the insult, then this principle (possibly biochemical in nature) must control cancer in all species.

This experiment has been made possible by grants from the Anna Fuller Fund and the Jane Coffin Childs Memorial Fund for Medical Research.

### REFERENCES.

AICHEL, O.—(1911) 'Ueber Zellverschmelzungen mit qualitativ abnormer Chromosomen verteilung als Ursachen des Geschwulstbil dung. Vorträge u. Auf. sätzeüber Entwicklungs mechanik.' Leipzig (W. Engelmann).
BAUER, K. H.—(1928) 'Mutations theorie der Geschwulstenstehung. Übergang von Körperzollen in Geschwulstzellen durch Genänderung.' Berlin (Springer).
*Idem*.—(1948) 'On the Cancer Problem.' Heidelberg (Springer),
BITTNER, J. J.—(1931) *Amer. J. Cancer*, **15**, 2202.
BOVERI, T.—(1929) 'The Origin of Malignant Tumors.' Translated by Marcella Boveri. Baltimore (The Williams & Wilkins Co.), p. 111.
CLOUDMAN, A. M.—(1932 ) *Amer. J. Cancer*, **15**, 568.—(1932*b*) *Genetics*, **17**, 468.
CURTIS, M. R., DUNNING, W. F., AND BULLOCK, F. D.—(1933) *Science*, **77**, 175.—(1934) *Amer. J. Cancer*, **21**, 86.
DAHLBERG, G.—(1940) *Upsala Läkareförenings förhandlingar. Ny fööjd.*, **76**, 21.
DUNNING, W. F., CURTIS, M. R., AND BULLOCK, F. D.—(1937) Occasional Publication, *Amer. Ass. Adv. Sci.*, 29.
EAST, E. M.—(1917) *Amer. Nat.*, **51**, 129.
FEDERLY, H.—(1936) *Hereditas*, **22**, 193.
LITTLE, C. C.—(1914) *Science*, **40**, 904.—(1934) *Amer. J. Cancer*, **22**, 578.—(1941) *J. nat. Cancer Inst.*, **2**, 133.
*Idem* AND STRONG, L. C.—(1924) *J. exp. Zool.*, **41**, 93.
LOCKHART-MUMMERY, J. P.—(1934) 'The Origin of Cancer.' London (J. & A. Churchill).
MURRAY, J. A.—(1908) *Sc. Rep. Imp. Cancer Res. Fund*, p. 69.
STARK, W. B., AND BRIDGES, C. B.—(1926) *Genetics*, **11**, 249.
STRONG, L. C.—(1926*a*) *Genetics*, **11**, 294.—(1926*b*) *J. exp. Med.*, **43**, 713.—(1929) *J. Cancer Res.*, **13**, 103.
*Idem* AND LITTLE, C. C.—(1920) *Proc. Soc. exp. Biol., N.Y.*, **18**, 45.
STURTEVANT, A. H.—(1948) *Amer. Sci.*, **36**, 225.
TYZZER, E. E.—(1916) *J. Cancer Res.*, **1**, 125.
VON HANSEMANN.—(1890) *Virchows Arch.*, **119**, 299.—(1891*a*) *Ibid.*, **123**, 352.—(1891*b*) *Ibid.*, **129**, 436.
WHITMAN, R. C.—(1919) *J. Cancer Res.*, **4**, 181.
WILSON, I. T.—(1924) *Genetics*, **9**, 343.